# Acute Tick-borne Rickettsiosis Caused by *Rickettsia sibirica* in the Russian Far East

**DOI:** 10.3201/eid1005.030437

**Published:** 2004-05

**Authors:** Oleg Y. Mediannikov, Yuri Sidelnikov, Leonid Ivanov, Eugenia Mokretsova, Pierre-Edouard Fournier, Irina Tarasevich, Didier Raoult

**Affiliations:** * Gamaleya Research Institute of Epidemiology and Microbiology, Moscow, Russia; † Far Eastern State Medical University, Khabarovsk, Russia; ‡ Université de la Méditerranée, Marseille, France; § Khabarovsk Plague Control Station of Ministry of Health of Russian Federation, Khabarovsk, Russia

**Keywords:** Rickettsia heilongjiangensis, Rickettsiaceae, spotted fever group rickettsiae, tick-borne diseases, Russia, Siberia

## Abstract

An acute tick-borne rickettsiosis caused by *Rickettsia heilongjiangensis* was diagnosed in 13 patients from the Russian Far East in 2002. We amplified and sequenced four portions of three rickettsial genes from the patients’ skin biopsy results and blood samples and showed that the amplified rickettsial genes belong to *R*. *heilongjiangensis*, which was recently isolated from *Dermacentor sylvarum* ticks in nearby regions of China. This rickettsia, belonging to subgroup of *R*. *japonica*, was previously suggested to be pathogenic for humans on the basis of serologic findings. We tested serum samples with different rickettsial antigens from 11 patients and confirmed increasing titers of immunoglobulin (Ig) G and IgM to spotted fever group rickettsiae, including *R. heilongjiangensis*. Clinical and epidemiologic data on these patients shows that this disease is similar to other tick-borne rickettsioses.

Russian Far East is a geographic, economic, and political unit within the Russian Federation. It consists of the smaller administrative areas (regions) located on or close to the Asian Pacific coast. The southern portion of Khabarovsk region, where this study was carried out, is situated alongside the Amur River down to the sea (Figure 1) and is characterized by peculiar combinations of subtropical and boreal biologic niches. Local experience suggests that tick-borne encephalitis, Siberian tick typhus, and, more recently, Lyme disease are prevalent in this territory, with marked seasonal disease peaks (*1*). In 2002, serologic evidence for acute granulocytic ehrlichiosis was found in the region (*2*).

**Figure 1:**
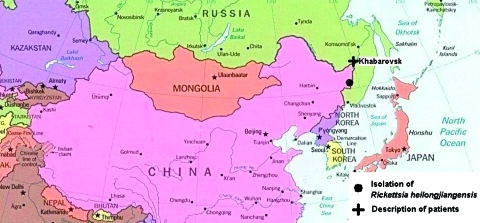
Geographic location of study area.

In 1932, a disease described as tick-borne fever (tick typhus) was identified in the Russian Far East (*3*). In Central Siberia in 1935, the agent of tick-borne fever, *Rickettsia sibirica*, was isolated and described (*4*–*6*), and several rickettsial isolates from the ticks from the Far Eastern Russia have also been identified as this new species (*7*). Since 1935, the cause of acute tick-borne spotted fever in the Russian Far East (including Khabarovsk Region) is thought to be *R*. *sibirica*, and the antigen of *R*. *sibirica* has been used for serologic studies in clinical laboratories. Nevertheless, differences between clinical pictures of tick-borne fever and differences in serologic cross-reactivity of sera from patients with tick-borne rickettsiosis have been reported in Siberia and the Russian Far East (*8*). *Dermacentor sylvarum* ticks have been identified as a vector. In 1990s, several strains of *R*. *sibirica* were also isolated from ticks in Primorye Region, south of Khabarovsk Region (*9*).

In Siberia and China, several new rickettsiae were found. *R*. *mongolotimonae* found in Inner Mongolia is closely related to *R*. *sibirica* (*10*) and causes acute disease in humans. Clinical cases have now been described in France (*11*,*12*). *R*. *heilongjiangensis* (strain 054) was first isolated from *D*. *sylvarum* ticks collected around Suifenhe in the Heilongjiang Province of China in 1982 (*13*). Serologic studies indicated that the organism was the probable cause of an acute tick-borne disease in humans (*14*). *R*. *hulinensis* (strain HL-93) was isolated in 1993 from *Haemaphysalis concinna* ticks collected in Hulin County, Heilongjiang Province (*10*). The pathogenicity of this agent in humans is unknown. By using molecular evolutionary genetic analyses, these rickettsiae were shown to form a well-defined clade distinct from other spotted fever group rickettsiae, including *R*. *japonica* (*15*). Both *D*. *sylvarum* and *H*. *concinna* ticks inhabit the Russian Far East, although *D*. *sylvarum* is quite rare (*1*). In this study, we identify the cause of an acute, febrile, tick-transmitted disease in the Russian Far East and describe the clinical picture in these cases.

## Patients and Methods

### Sample Collection

During this study in 2002, we screened almost all patients who were suspected clinically to have a tick-borne rickettsiosis and who were hospitalized in the infectious diseases department of Khabarovsk Municipal Hospital No.10. Five to 20 days before onset of the disease, most patients reported tick bites or tick exposure. Some had typical eschars on the skin without a history of a tick bite. The illness was acute, febrile, and usually involved a macular or maculopapular rash. All patients were successfully treated with a 14-day course of doxycycline. Patients with clinically evident features of tick-borne encephalitis (neurologic signs) or Lyme disease (erythema migrans) were excluded from the study. In total, samples from 65 patients were investigated, 64 blood samples and 17 skin biopsy results. Paired sera from most of the patients were tested with conventional serologic methods. The first serum sample was taken immediately after admission to the hospital, usually 1–9 days after the onset of the disease. The second serum sample was taken 4–18 days later.

### DNA Extraction, PCR, and Sequencing

Buffy coats were separated from fresh blood specimens immediately after collection at patient’s admission. DNA was extracted from the buffy coat and skin biopsies samples with QIAmp DNA Blood Mini Kit and DNeasy Tissue Kit (Qiagen, Tokyo, Japan) according to manufacturer’s instructions.

Oligonucleotide primers used in this study and annealing temperatures are shown in Table 1. We used the nested polymerase chain reaction (PCR) method for primary detection of the rickettsial DNA in human samples. The *gltA* (citrate synthase) gene was chosen as the target for amplification because of its genus specificity and conservativeness. The primer CS1d (*16*), which is used traditionally to amplify the full-length *gltA* gene, is complementary to the ultimate 5′-end of the open reading frame for this gene of *R*. *prowazekii*. Since we expected to find DNA of the spotted fever group rickettsiae, we modified the primer to be completely homologous to the corresponding portion of the gene of *R*. *conorii* (Table 1). *R*. *conorii* has been chosen as a template to design primer CS2d as the only spotted fever group rickettsia having a completely sequenced genome at the time of investigation. Comparison of these two primers indicated that the newly designed primer (CS2d) was at least 100 times more sensitive than CS1d in the amplification of the serially diluted DNA of *R*. *montanensis*, which was used as a positive control (data not shown). Primers CS2d and CSendR amplify the full-length of the *gltA* gene. Primers CS877f and CS1258r were used in the nested PCR assay. To amplify the full-length of the *gltA* gene, we used PCR followed by hemi-nested PCR using the product of the first PCR as a template. Two conservative regions of the outer membrane protein A (*ompA*) gene were amplified (base positions 91–680 and 3,608–6,789 with respect to the sequence published for *R*. *rickettsii*) by using previously described primers (*17*). Initial amplification of the 3,182-bp longer region was achieved with primers 190-3588f and 190-5044r and with primers 190-4859f and 190-6808r. Products of this reaction were used later in the nested and hemi-nested PCR reactions with corresponding primers. The *ompB* gene was amplified from clinical samples and DNA by using standard primers (*18*), except for the ultimate forward primer 120-M59, which was found to produce nonspecific amplicons with the human DNA in clinical samples. Instead, for clinical specimens, we used a newly designed primer, 120-M36. As with the *ompA*, the DNA from clinical samples was amplified in two steps. In the first step, we amplified two halves of the gene, with a small overlapping region with primers 120-M36 with 120-2988 and 120-2788 with 120-4879. The products of these reactions were used as templates for each of the specific nested or heminested reactions for seven regions. All primers were purchased from Eurogentec (4102 Seraing, Belgium).

**Table 1:** List of the primers used to detect rickettsial DNA^a^

Gene for amplification	Primer name	Primer sequence 5′-3′	Annealing temperature
*gltA*	CS1d	ATGAC**T**AATG**GC**AATAATAA	
	CS2d	ATGAC**C**AATG**AA**AATAATAA**T**	50°C
	CSEndr	CTTATACTCTCTATGTACA	
	RpCS877p	GGGGACCTGCTCACGGCGG	54°C
	RpCS1258n	ATTGCAAAAAGTACAGTGAACA	
*ompA*, 5′- portion	190-70	ATGGCGAATATTTCTCCAAAA	53°C
	190-701	GTTCCGTTAATGGCAGCATCT	
*ompA,*3′- portion	190-3588f	AACAGTGAATGTAGGAGCAG	46°C for the first round 50°C for the nested and heminested rounds
	190-3968r	TAGCAGCTGATTTAGTAGCT	
	190-4084f	CATCACCGATATTTCTAGC	
	190-4338f	TTCAGGAAACGACCGTACG	
	190-4406r	ACTATACCCTCATCGTCATT	
	190-4859f	GCGAAATCCAAGGTACAGG	
	190-5044r	AACTTGTAGCACCTGCCGT	
	190-5125f	GCGGTTACTTTAGCCAAAGG	
	190-5238r	ACTATTAAAGGCTAGGCTATT	
	190-5768f	CACCGCTACAGGAAGCAGAT	
	190-5831r	GTGTCGCTAGGTTTTACAAC	
	190-6228f	CGTTGAAGTATAGCATC	
	190-6427r	ATCTAAGCCCAGCTAGCGGT	
	190-6808r	CACGAACTTTCACACTACC	
*ompB*	120-M36	TTCTACAGCTACCATAGTAGCCA	50°C
	120-607	AATATCGGTGACGGTCAAGG	
	120-807	CCTTTTAGATTACCGCCTAA	
	120-1378	TAAACTTGCTGACGGTACAG	
	120-1497	CCTATATCGCCGGTAATT	
	120-2113	CGATGCTAACGTAGGTTCTT	
	120-2339	CTTGTTTGTTTAATGTTACGGT	
	120-2778	AAACAATAATCAAGGTACTGT	
	120-2988	CCGGCTATACCGCCTGTAGT	
	120-3462	CCACAGGAACTACAACCATT	
	120-3599	TACTTCCGGTTACAGCAAAGT	
	120-4232	GGTTTCTCATTCTCTCTATATGG	
	120-4346	CGAAGAAGTAACGCTGACTT	
	120-4879	TTAGAAGTTTACACGGACTTTT	

^a^Differences between primers CS1d and CS2d are indicated by bold letters.

Amplification has been carried out by routine methods in a final volume of 50 μL with appropriate negative controls (*10*,*17*). In all reactions, as a positive control, we used a mixture of DNA of the following microorganisms: *Anaplasma phagocytophilum*, *Neorickettsia sennetsu*, *Wolbachia pipientis*, *Francisella tularensis*, *Bartonella henselae*, *Borrelia garinii*, *Coxiella burnetii*, and *R*. *montanensis*. We found that the cocktail of DNA of tick-borne or phylogenetically close to tick-borne bacteria is convenient to use in PCR with potentially polyinfected human samples.

We checked all samples for other possible tick-borne bacterial and pathogens with the following primers under conditions suggested in published references: SL primers for *ospA* gene of *Borrelia* (*19*); BhCS.781p and BhCS.1137n primers for *Bartonella* citrate synthase (*20*); HE1, HE3, and HE4 primers for 16S rDNA of *Ehrlichia chaffeensis* (*21*); GE3A, GE10r, GE9f, and GE2 primers for 16S rRNA gene of *A*. *phagocytophilum* (*22*); and P3708 and p4257 primers for the gene encoding p44 protein *of A. phagocytophilum* (*23*) [data not shown]. No positive results have been obtained among patients described here.

The PCR products were purified for DNA sequencing with the QIAquick PCR purification kit (Qiagen) and then directly sequenced by using PCR primers. Sequencing reactions were carried out with a D-rhodamine terminator cycle DNA sequencing kit (Applied Biosystems, Foster City, CA) according to the manufacturer’s instructions. Sequencing reaction products were resolved by electrophoresis with an ABI Prism 377 sequencer (Applied Biosystems). The results obtained were processed into sequence data with AutoAssembler software (Applied Biosystems). The sequences of the *gltA*, both regions of the *ompA* gene, and the *ompB* genes were aligned by using the software Genetix-Win 5.1 (Software Development Co., Ltd., Japan). Sequences in the *ompB* genes of *R*. *heilongjiangensis* and *R*. *hulinensis* were not available in the GenBank database so we amplified and sequenced the gene. The sequences used for comparison were obtained from the GenBank database, aligned, and then corrected manually to preserve codon alignment and conserved motifs. Sites with ambiguous alignments were removed before phylogenetic analysis. The phylogenetic tree was calculated by using neighbor-joining method with MEGA2 Version 2.1 software (available from: http://megasoftware.net). Internal node support was verified by using the bootstrap method with 100 replicates.

### Serologic Studies

Two serologic tests were performed in Khabarovsk Plague Control Station: immunofluorescence studies with a combined antigen consisted of two local strains of *B. garinii* and one local strain of *B. afzelii*, and an enzyme-linked immunosorbent assay detected antibodies against tick-borne encephalitis virus (*24*). For further investigations, sera were dried on blotting papers as described previously (*25*) and transported to Marseilles, where microimmunofluorescence testing (*26*) was performed by using in-house prepared antigens of *R*. *heilongjiangensis* (strain 054, ATCC VR-1524), *R*. *hulinensis* (strain HL-93, ATCC VR-1527), *R*. *sibirica* (strain 246, ATCC VR-151), *R*. *conorii* (strain Moroccan ATCC VR-141), *C*. *burnetii* (strain Nine Mile, ATCC VR-615), *Orientia tsutsugamushi* (strains Gilliam, Karp, Kato, and Kawazaki), *E*. *chaffeensis* (strain Arkansas), *A*. *phagocytophilum* (strain Webster), *Bartonella henselae* (Houston-1, ATCC-49882), and *B*. *quintana* (strain Oklahoma). Antigens were applied by pen point to 18-well microscope slides, dried for 30 min, and fixed. Appropriate positive- and negative-control serum samples were tested on each slide together with twofold dilutions of patients’ serum samples made in 3% nonfat dry milk in phosphate-buffered saline (PBS). Slides were incubated in a moist chamber for 30 min at 37°C, washed twice in PBS and once in distilled water (10 min each); reactive antibodies showed fluorescein isothiocyanate–conjugated goat anti-human γ chain and μ-chain immunoglobulins (BioMérieux, Marcy l’Etoile, France). After the conjugate was added, slides were incubated for 30 min at 37°C, washed in two PBS for 10 min and for 5 min in distilled water, and mounted in buffered glycerol. Endpoints for each antigen were the lowest concentrations of serum that definitely conferred fluorescence on bacteria.

### Nucleotides Accession Numbers

Nucleotide sequences obtained during this study were deposited in GenBank under the following numbers: AY260451 for *ompB* gene of *R*. *heilongjiangensis*, strain 054; AY260452 for *ompB* gene for *R*. *hulinensis*; AY280712 for *ompB* gene; AY280711 for previously tandemly repeated region portion of *ompA* gene; AY280710 for another portion; and AY280709 for *gltA* gene of *Rickettsia* spp. found in this study.

## Results

We amplified and sequenced DNA of *R*. *heilongjiangensis* in samples from 16 patients. Serum samples from 11 were available for serologic studies, and clinical and epidemiologic data have been analyzed for 13 patients, including all patients with investigated serum samples.

Ten of 17 samples of DNA extracted from skin eschars and seven of 64 samples of DNA extracted from buffy coats were positive in the nested PCR for the *gltA* gene. In one patient, both the skin biopsy and the buffy coat were positive and had the same DNA sequence. Because we had limited amounts of extracted DNA, we attempted to amplify both the *ompA* and *ompB* genes from six samples (three skin biopsies and three blood samples), which were previously positive in nested PCR with primers for citrate synthase gene. All positive samples were also screened by PCR for other possible bacterial tick-borne pathogens and were found to be negative. Results from testing, serologic or PCR, that suggested double infection were excluded from the study. Clinical picture of the disease was analyzed in patients with *R*. *heilongjiangensis* infection to describe the disease associated with this organism.

**Figure 2:**
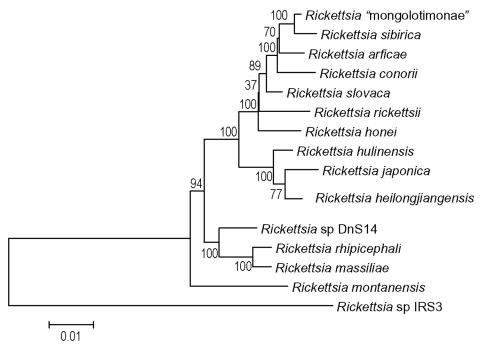
Phylogenetic tree, representing position of *Rickettsia heilongjiangensis*. The tree was made using MEGA 2.1 software after alignment of both portions of *ompA* genes obtained from GenBank and sequenced during this study by GENETIX-WIN 5.1 software. The scale represents a number of substitutions per base per indicated horizontal distance. The numbers present at nodes of the tree represent the number of bootstrap replicates of 100 that display the indicated sequence groupings.

All 17 nested PCR amplicons of amplified *gltA* gene were directly sequenced and showed 100% homology. Six amplicons of *ompA* and *ompB* genes of corresponding samples were also identical with each other. We obtained full-length *gltA* gene sequence, 590 bp and 3,182 bp (excluding primer sequences) of 5′- and 3′-regions of the *ompA* gene, respectively, and a 4,852-bp length sequence of the *ompB* gene. A BLAST search showed that all sequences were completely homologous to correspondent genes of *R*. *heilongjiangensis*. Figure 2 shows the phylogenetic relationships of this *Rickettsia* and other species based on the analysis of both concatenated portions of the *ompA* gene.

Serum samples from 11 of 16 patients were studied; data are presented in Tables 2 and 3. None of the samples had serologic evidence of any other nonrickettsial acute, tick-borne disease. One previously vaccinated patient had a low titer of immunoglobulin (Ig) G against tick-borne encephalitis virus, and two had low titers to both *B*. *henselae* and *B*. *quintana* (data not shown)*.* In 9 of 11 available paired sera from PCR-positive patients, we found serologic evidence for acute rickettsial infection, e.g., the presence of IgM antibodies, seroconversion, or a fourfold rise in antibody titer against rickettsial antigens. In samples from two patients, IgG and IgM antibody titers to *R*. *heilongjiangensis* were highest. In samples from five patients, the same titers were present against *R*. *conorii* and *R*. *heilongjiangensis*. In samples from one patient, we found the same levels of antibodies to all rickettsial antigens. Levels of antibodies against *R*. *sibirica* in all cases, except one, were lower than against *R*. *heilongjiangensis.* In four of nine cases, titers were identical against antigens of *R*. *hulinensis* and *R*. *sibirica.* Absence of specific antibodies against rickettsiae in patients 2 and 3 could be explained either by early treatment with a specific antimicrobial drug or probable errors in serum sample collection and treatment.

**Table 2:** Serologic data of 11 patients with *Rickettsia* infection^a^

Patient no.	Disease onset date	Antimicrobial treatment^b^	PCR for		Sera	Collection date	IgM tests				
			*gltA* gene	*ompA* and *ompB* genes							
							*R. heilongjiangensis*	*R. hulinensis*	*R. sibirica*	*R. conorii*	
1	6/18	6/23	+	NI	1	6/24	0	0	0	0	
					2	6/28	1/32	1/32	1/32	1/32	
2	6/20	6/23	+	+	1	6/24	0	0	0	0	
					2	7/9	0	0	0	0	
3	6/21	6/24	+	NI	1	6/5	0	0	0	0	
					2	7/12	0	0	0	0	
4	6/22	6/26	+	NI	1	6/26	1/32	1/16	1/16	1/32	
					2	7/1	1/64	1/64	1/64	1/64	
5	6/22	6/28	+	NI	1	6/28	0	0	0	0	
					2	7/16	1/128	1/32	1/32	1/128	
6	6/29	7/2	+	+	1	7/3	0	0	0	0	
					2	7/8	1/64	1/64	1/64	1/64	
7	6/30	7/4	+	NI	1	7/5	0	0	0	0	
					2	7/16	1/1,024	1/1,024	1/256	1/1,024	
8	7/1	7/8	+	NI	1	7/8	0	0	0	0	
					2	7/26	1/512	1/126	1/126	1/512	
9	7/6	7/12	+	NI	1	7/15	1/64	1/64	1/64	1/64	
					2	7/31	1/128	1/128	1/128	1/128	
10	7/23	7/28	+	+	1	7/29	0	0	0	0	
					2	8/13	1/32	1/32	1/16	1/16	
11	7/25	8/1	+	NI	1	8/2	1/256	1/256	1/64	1/64	
					2	8/15	1/256	1/256	1/64	1/64	

^a^PCR, polymerase chain reaction; Ig, immunoglobulin; NI, not investigated. ^b^Date of beginning of antimicrobial drug therapy.

**Table 3:** Serologic data of 11 patients with *Rickettsia* infection^a^

Patient no.	Disease onset date	Antimicrobial treatment^b^	PCR		Sera	Collection date^g^	IgG tests				
			*gltA* gene	*ompA* and *ompB* genes							
							*R. heilongjiangensis*	*R. hulinensis*	*R. sibirica*	*R. conorii*	
1	6/18	6/23	+	NI	1	6/24	0	0	0	0	
					2	6/28	1/1024	1/512	1/512	1/1,024	
2	6/20	6/23	+	+	1	6/24	0	0	0	0	
					2	7/9	0	0	0	0	
3	6/21	6/24	+	NI	1	6/5	0	0	0	0	
					2	7/12	0	0	0	0	
4	6/22	6/26	+	NI	1	6/26	1/64	1/64	1/64	1/64	
					2	7/1	1/2,048	1/512	1/512	1/2048	
5	6/22	6/28	+	NI	1	6/28	0	0	0	0	
					2	7/16	1/128	1/128	1/128	1/128	
6	6/29	7/2	+	+	1	7/3	0	0	0	0	
					2	7/8	1/128	1/64	1/64	1/128	
7	6/30	7/4	+	NI	1	7/5	0	0	0	0	
					2	7/16	1/256	1/128	1/256	1/256	
8	7/1	7/8	+	NI	1	7/8	0	0	0	0	
					2	7/26	0	0	0	0	
9	7/6	7/12	+	NI	1	7/15	1/1,024	1/1,024	1/1,024	1/1,024	
					2	7/31	1/1,024	1/1,024	1/1,024	1/1,024	
10	7/23	7/28	+	+	1	7/29	0	0	0	0	
					2	8/13	1/1,024	1/1,024	1/256	1/256	
11	7/25	8/1	+	NI	1	8/2	1/256	1/256	1/128	1/128	
					2	8/15	1/256	1/256	1/128	1/128	

^a^PCR, polymerase chain reaction; Ig, immunoglobulin; NI, not investigated. ^b^Date of beginning of antimicrobial drug therapy.

**Figure 3:**
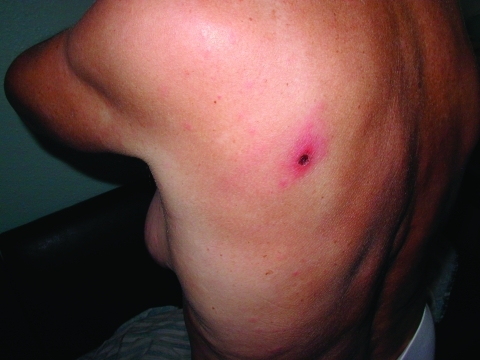
Eschar and faint macular rash in patient 9.

Epidemiologic, clinical, and laboratory data available in 13 of 16 PCR-positive patients are given in Table 4. Before the onset of the disease in the summer 2002 (from June to August), all patients had a history of tick bite, tick exposure, or a stay in an epidemiologically suspected location. After an incubation period of 4 to 7 days, the patient had a sudden onset with fever, but no specific symptoms appeared during the first several days. In 12 patients, a macular or maculopapular rash appeared but was usually faint. Twelve patients had a primary lesion (eschar) at the site of tick attachment (Figure 3). The eschar consisted of a necrotic central region (50–150 mm in diameter) surrounded by infiltrated and inflamed tissue (70–400 mm in diameter), and a zone of hyperemia (250–500 mm in diameter). The eschars were found on the waist and buttocks region (four); lower extremities (two); upper extremities and axillar region (two); and back, chest, neck, and abdomen (one case each). In two patients, we noticed subcutaneous lymphangitis and regional lymphadenopathy. Initial conventional treatment at home, with a combination of antipyretics, analgesics, and antibacterial therapy (oral penicillins) did not result in improvement; 5–9 days after the onset of symptoms, all patients were admitted to the hospital. They received oral doxycycline for 14 days and antihistamine therapy, and clinical symptoms resolved within 2 to 3 days. Laboratory tests in the hospital showed elevated levels of serum transaminases (alanine aminotransaminase and aspartate aminotransaminase) in 46% and 15% of patients, respectively, and these remained elevated even during convalescence but were normal at follow-up at 3 to 4 weeks.

**Table 4:** Epidemiologic, clinical and laboratory data of 13 patients with rickettsiosis.

Feature or sign	Value (n = 13)
Sex, male/female	8/5
Age, y, mean	52 (18–66)
Mean period between onset and hospitalization, d	4.6
Mean stay at the hospital, d	5.7
Primary diagnosis of rickettsiosis at admission	9
History of tick bite	6
Incubation period, d, median (range)	5.5 (4–7)
Antibiotics taken before hospitalization	2
Chills	13
Malaise	13
Headache	13
Dizziness	11
Myalgias, arthralgias	13
Nausea	2
Anorexia	13
Maculopapular rash	12
Rash appearance after onset of disease, d, median	3.6
Duration of rash, d, median (range)	5.5 (4–7)
Presence of eschar	12
Lymphadenopathy regional to the eschar	10
Subcutaneous lymphangitis, leading to regional lymph nodes	2
Hepatomegaly	5
Splenomegaly	2
Sleep disturbances	7
Leukocytosis at admission, (>9,000/mm^3^)	6
Leukopenia at admission, (<4,000/mm^3^)	2
Increased ESR (>15 mm/h for men, >20 mm/h for women)	12
Thrombocytopenia, (<150,000/mm^3^)	3
Proteinuria (>0.033 g/L)	1
Increased AlAT activity, >1.5 times	6
Increased AsAT activity, >1.5 times	2
Doxycycline treatment, 100 mg twice daily for 14 d	13

^a^ESR, erythrocyte sedimentation rate; A1AT, alanine aminotransferase; AsAT, aspirate aminotransferase.

## Discussion

Our findings suggest that patients had an acute infection with *R*. *heilongjiangensis*. Clinical data were typical for acute rickettsial infections. We successfully amplified four portions of three different rickettsial genes from human blood and skin samples, which suggested the presence of this microorganism. We did not amplify any of these genes in samples from healthy donors or from patients suffering from other infectious diseases (negative controls). The *gltA* gene is conservative, especially among *Rickettsia* species (*16*), and *ompA* and *ompB* genes encode main surface proteins of rickettsiae. These gene sequences were completely homologous to the spotted fever group *R. heilongjiangensis*. Previous GenBank sequences were recently corrected (Fournier PE et al., unpub. data). The identity of sequenced PCR products among patients, clinical data, and epidemiologic data suggest the focality of this rickettsiosis.

The serologic data support PCR and sequencing findings. We showed that *Rickettsia* produces a clear immunologic response in patients. We studied paired sera from patients for reactivity with antigens of several species in the *Rickettsia* genus, including *R*. *heilongjiangensis* and *R*. *hulinensis*. Seroconversion, presence of IgM, or increasing antibody titers were observed in most patients. In seven cases, sera reacted at higher titers with antigen of *R*. *heilongjiangensis* when compared with *R*. *sibirica*, the only currently identified tick-borne rickettsia in the Russian Far East. In 77%, the titers were identical against *R*. *heilongjiangensis* and *R*. *conorii* antigens*.* Although serologic cross-reactions are common among rickettsiae of the spotted fever group (*26*), the finding of lower titers against the phylogenetically more closely related *R*. *hulinensis* than the relatively more distant *R*. *conorii* was unexpected.

Epidemiologic evidence of tick bite or exposure, rash, primary lesion (eschar) at the site of tick bite, and rapid recovery after doxycycline treatment support a rickettsial cause for the disease. Some peculiarities were noticed when signs were compared with infection caused by *R*. *sibirica* in the Central Siberia. Seasonal peak of infections is in the end of June and July. For Siberian tick typhus, the seasonal peak is the end of April and May. The rash that accompanies tick-borne rickettsiosis in the Russian Far East is less obvious, and the disease apparently affects older people than Siberian tick typhus. Only 1 of 13 patients was >45 years of age. Generally, the disease is mild, with no serious complications or death recorded.

The epidemiology of the disease remains mostly unknown. Recently, DNA of the *Rickettsia* described in our report was amplified from *H*. *concinna* ticks collected in Siberia (S. Shpynov, unpub. data).

PCR-based technologies and direct sequencing provide a fast and precise diagnosis or rickettsiosis. The preferable method may be PCR on eschar biopsy samples because this technique has high sensitivity and probability of finding rickettsial DNA (*27*). Serologic studies of samples from Russian Far East area should include tests with antigens of *R*. *heilongjiangensis*.

Results of our studies showed that acute febrile tick-borne disease caused by *R*. *heilongjiangensis* is prevalent in the Russian Far East. Molecular biology approaches enabled us to identify the cause of an acute disease and to detect its bacterial origin. As no evidence of *R*. *sibirica* human infection was found in our study, further investigations are needed clarify its role in human pathology in the Russian Far East, especially the Khabarovsk Region.
